# Harnessing Plant–Microorganism Interactions to Mitigate Biotic and Abiotic Stresses for Sustainable Crops

**DOI:** 10.3390/plants15040647

**Published:** 2026-02-19

**Authors:** Mayara Santana dos Santos, Silas Pessini Rodrigues, Adriana Silva Hemerly, Antonio Alberto Ribeiro Fernandes, Patricia Machado Bueno Fernandes

**Affiliations:** 1 Núcleo Multidisciplinar de Pesquisa em Biologia, Universidade Federal do Rio de Janeiro, Rio de Janeiro 25.240-005, Brazil; contatomayaras@gmail.com (M.S.d.S.); srodrigues@xerem.ufrj.br (S.P.R.); 2 Instituto de Bioquímica Médica Leopoldo de Meis, Universidade Federal do Rio de Janeiro, Rio de Janeiro 21.941-902, Brazil; hemerly@bioqmed.ufrj.br; 3 Núcleo de Biotecnologia, Universidade Federal do Espírito Santo, Vitória 29.043-215, Brazil

**Keywords:** bio-inoculants, induced systemic resistance, genetic engineering, sustainable biotechnology

## Abstract

Climate change has intensified the occurrence of biotic and abiotic stresses, representing a major threat to agricultural productivity. This climate variability, coupled with the excessive use of agrochemicals, not only compromises environmental sustainability but also exacerbates food insecurity, directly affecting food availability and quality. In this context, biotechnological strategies have proven essential for mitigating the effects of stress on plants, promoting practices focused on agricultural sustainability. Notable among these strategies is the use of plant growth-promoting microorganisms, which are emerging as promising alternatives capable of improving plant tolerance to stress conditions and simultaneously reducing dependence on agrochemicals. These microorganisms can act as nitrogen fixers and solubilizers of nutrients, such as phosphorus and potassium. Additionally, they can influence plant immune responses by inducing systemic resistance and promoting the synthesis of phytohormones, such as auxins, cytokinins, and abscisic acid, which support plant development during the stress response. The interaction between plants and microorganisms represents a sustainable agricultural management strategy capable of enhancing crop tolerance to environmental adversities. In this review, we discuss the microorganisms known to establish beneficial interactions with plants, leading to improved performance under biotic and abiotic stress. Overall, this work highlights the potential of plant–microbe partnerships as a cornerstone for advancing sustainable agriculture in the face of global challenges.

## 1. Introduction

Climate variability, population growth, and food security have been widely debated and investigated by the scientific community, given their significant impact on sustainable and social development [1,2]. It is estimated that by 2050, the global population will surpass 9.7 billion people, leading to a 60% increase in food demand [3,4]. In the same year, the average global temperature is expected to rise by 1.5 °C because of anthropogenic actions that contribute to global warming and climate change. This climatic variability is a factor that exacerbates world hunger, particularly in low-income countries, where resources are scarce and access to technology is limited [5,6]. These future projections directly affect agricultural production and intensify the search for ecologically sound solutions aligned with the United Nations (UN) Sustainable Development Goals (SDGs) [7,8]. To achieve sustainable and efficient agricultural production, it is essential to reduce the impact of plant diseases and promote plant growth adapted to diverse climatic conditions. In this context, sustainable strategies capable of mitigating biotic and abiotic stresses have gained increasing relevance, particularly those involving beneficial plant–microorganism interactions. At the same time, smallholder farming, responsible for supplying more than half of the world’s food, faces growing challenges associated with climate variability, declining soil fertility and limited financial resources, making the development of accessible biological solutions even more urgent. These factors collectively inhibit crop development, diminish productivity, and endanger the livelihoods of populations. Confronting these challenges necessitates the adoption of sustainable and ecological alternatives to costly and environmentally harmful agrochemicals [9].

Interactions between plants and microorganisms are fundamental to the health and productivity of plant ecosystems. Traditionally, this relationship was viewed from a dichotomous perspective, focusing primarily on pathogens or specific symbionts. However, the contemporary paradigm recognizes the plant as a ‘holobiont’, a complex entity whose function is intrinsically linked to its associated microbial community, the microbiome [10]. This microbiome can be influenced by biotic and abiotic factors, such as temperature, soil pH, nutrient availability, and interactions with other microorganisms [11]. Among these interactions, commensal relationships, whether symbiotic or mutualistic, facilitate exchanges that benefit both partners without impairing their development [12,13]. In this context, plant growth-promoting microorganisms (PGPMs) facilitate plant development by mobilizing key nutrients, such as phosphate and nitrogen, producing phytohormones, including auxins and cytokinins (CK), and synthesizing a variety of organic and inorganic compounds [14,15]. PGPMs can be classified as endophytic, when they colonize internal plant tissues and contribute to the production of secondary metabolites, hormones, and the induction of resistance to biotic and abiotic stresses, or as epiphytic, when they reside on the plant surface and protect against pathogens through the production of antimicrobial compounds that modulate microbiota balance [16,17]. Commensal or neutral relationships may become pathogenic depending on the host, environmental conditions, and nutrient availability. While beneficial interactions exist, certain microorganisms establish pathogenic relationships with plants, triggering biotic stress responses that affect host metabolism and limit growth and productivity [11].

Research has elucidated that plants engage in complex molecular communication with their associated microbial communities, including both the rhizosphere and the phyllosphere. Through the release of diverse root exudates and leaf-derived metabolites, the host actively shapes the structure of its microbiome by recruiting beneficial microorganisms that contribute to pathogen suppression, improved nutrient acquisition, and overall growth promotion [18]. These interactions extend throughout the plant’s below- and above-ground interfaces, where microbial partners play critical roles in modulating resistance to biotic and abiotic stresses, thereby supporting plant fitness under variable environmental conditions [19]. Given the considerable productivity losses caused by these biotic and abiotic stressors, with approximately 30% of global agricultural production loss attributed to abiotic stresses and 21 to 30% to biotic stresses [20,21], identifying strategies that support plant resilience has become increasingly urgent.

The development of biotechnological strategies for formulating PGPMs has emerged as a promising, sustainable alternative to synthetic pesticides. For instance, the application of diazotrophic microorganisms capable of fixing atmospheric nitrogen into plant-available forms can reduce the reliance on synthetic fertilizers while simultaneously promoting plant development and supporting the mitigation of both biotic and abiotic stresses [22]. The adoption of PGPMs aligns with the broader framework of sustainable agriculture, which advocates environmentally responsible approaches as alternatives to the intensive use of conventional agrochemicals that are often formulated with compounds potentially harmful to living organisms and the environment. Although agrochemicals play an essential role in agricultural productivity by stimulating plant growth through fertilizers that supply essential nutrients, their excessive use entails significant risks [23]. These risks range from cardiorespiratory diseases associated with direct or indirect exposure to such substances to severe ecological disturbances, including increased bee mortality, a phenomenon with critical implications for ecosystem stability [24,25]. Driven by global population growth, the consumption of mineral fertilizers has also increased continuously and substantially. In 1961, before the Green Revolution, global consumption reached 30.85 million tons and rose to 88.54 million tons by 2019. Specifically, the use of nitrogen fertilizers increased by 250 percent between 1969 and 2019. Among the primary macronutrients, nitrogen, phosphorus, and potassium, nitrogen-based fertilizers remain the most widely applied [24].

In light of these environmental, agronomic and health concerns, as well as the growing interest in sustainable microbial-based technologies such as PGPMs, a deeper understanding of the mechanisms governing plant–microbe interactions becomes essential for advancing practical and scalable solutions in modern agriculture [26]. This review integrates recent investigations linking microbial action mechanisms to plant phenotypic responses under biotic and abiotic stress. We discuss beneficial plant-microbe interactions. Furthermore, we explore the potential of engineered microorganisms in mitigating plant stress. Central to this discussion is the essential challenge of translating this mechanistic and ecological knowledge into practical, sustainable applications. Consequently, the strategic manipulation of the plant microbiome emerges as a cornerstone for a new agricultural paradigm, aiming to develop crops that optimally manage their microbial communities or to deploy next-generation microbial consortia as advanced bioinoculants. Ultimately, this review highlights the potential of plant-microbe partnerships as a resilient foundation for sustainable agriculture in the face of global environmental challenges [27,28,29,30,31,32,33,34].

## 2. Classical Mechanisms of Plant Growth-Promoters

Beneficial microorganisms associated with plants, particularly plant growth-promoting rhizobacteria (PGPR) and plant growth-promoting fungi (PGPF), modulate plant performance through a constellation of metabolic, signaling, and physiological processes that extend far beyond the traditional view of nutrient mobilization. Rather than acting through a single dominant pathway, these microorganisms influence plant growth and stress resilience through integrated networks of nutrient flux, hormonal regulation, redox balance, and gene expression [35,36,37].

Importantly, the magnitude, coordination, and ecological relevance of microbial-mediated processes are not intrinsic or universally expressed properties of PGPR and PGPF, but are strongly shaped by environmental context [38]. Accumulating evidence demonstrates that abiotic factors such as soil nutrient status, pH, temperature, water availability, and salinity critically modulate microbial metabolism, signaling capacity, and plant responsiveness, thereby altering the balance among nutrient mobilization, hormonal crosstalk, and downstream physiological outcomes [39]. In parallel, climate change, associated abiotic stresses directly affect beneficial microorganisms themselves, influencing their survival, metabolic activity, gene expression profiles, and community dynamics within soil and rhizosphere environments. Experimental studies show that drought, heat, and salinity induce pronounced shifts in microbial community composition and functional activity, accompanied by stress-specific transcriptional reprogramming related to carbon metabolism, osmoprotection, and stress tolerance [40,41]. Together, these environmental pressures drive both short-term environmentally regulated microbial functions and longer-term adaptive responses in plant growth-promoting microorganisms, ultimately impacting their persistence, consistency, and efficacy as inoculants under field conditions [38]. Within this framework, the mechanisms detailed below should be interpreted as interconnected and environmentally modulated processes, whose effectiveness depends on local abiotic constraints and the plant’s developmental context.

### 2.1. Nutrient Mobilization and Root-System Remodeling

The PGPR and PGPF reshape the rhizosphere through metabolic activities that enhance the bioavailability of key nutrients. Bacterial communities mobilize nitrogen via biological nitrogen fixation, converting atmospheric N_2_ into ammonium and subsequently into nitrite and nitrate, which integrate into plant nitrogen metabolism [42,43]. Furthermore, they enhance nutrient availability through several key mechanisms: phosphate solubilization mediated by the secretion of organic acids (e.g., by *Bacillus* spp.), iron acquisition via siderophore production which supports critical processes like chlorophyll biosynthesis and the enzymatic degradation of complex organic matter by genera such as *Paenibacillus* and *Streptomyces*, thereby enriching soil fertility through the release of simpler compounds [36,44]. These microbial activities remodel root architecture through signaling molecules that stimulate root elongation, lateral root formation, and increased root hair density, responses observed in both [45] and PGPF [46]. Effective root colonization is ensured by traits such as biofilm formation via exopolysaccharide production and motility guided by chemotaxis toward root exudates, which together enhance persistence in the rhizosphere. Finally, the remarkable adaptability of these bacteria is supported by horizontal gene transfer, enabling the rapid acquisition of genes associated with stress tolerance, nutrient acquisition, and contaminant degradation, which is essential for survival and functionality in challenging and fluctuating environments [47].

### 2.2. Hormonal Modulation and Growth Regulation

Classical plant hormones form a second major axis of PGPR/PGPF action. Many PGPR synthesize auxins or indole-3-acetic acid (IAA), CK, gibberellins (GA), and abscisic acid (ABA), influencing cell division, elongation, and differentiation in both shoots and roots [48,49]. Fungal partners also contribute to phytohormone pools, including IAA, GA, and CK, supporting vegetative development and helping plants maintain growth under stress [11,37]. Through these hormonal adjustments, microbial partners stabilize key developmental processes even when plants face drought, salinity, temperature stress, or pathogen attack.

In parallel, these microorganisms contribute to plant tolerance to abiotic stresses by producing ACC deaminase, thereby lowering stress induced ethylene (ET) levels, synthesizing osmoprotectants such as proline and glycine betaine that support cellular homeostasis under saline conditions, and inducing plant antioxidant systems, including catalase and superoxide dismutase, to mitigate oxidative damage [50].

### 2.3. Redox Homeostasis, Osmoprotection, and Stress Buffering

One of the hallmark features of PGPR and PGPF activity is their capacity to modulate interconnected biochemical, osmotic, and redox-regulatory systems that sustain cellular homeostasis under stress conditions. Under osmotic or oxidative stress, PGPR promote the accumulation of key osmoprotectants, including proline, glycine betaine, and soluble sugars, which play central roles in osmotic adjustment, stabilization of membranes and proteins, and preservation of cellular hydration status [51,52]. Proline additionally functions as a molecular chaperone and reactive oxygen species (ROS) scavenger, linking osmoprotection directly to redox balance, while soluble sugars contribute to both carbon buffering and signaling during stress. In parallel, these microorganisms enhance the plant antioxidant machinery by upregulating enzymes such as catalase (CAT), peroxidase (POD), glutathione peroxidase (GPX), and superoxide dismutase (SOD), thereby limiting excessive ROS accumulation and preventing oxidative damage to lipids, proteins, and nucleic acids [35]. Similar mechanisms are observed in PGPF, which stimulate the accumulation of osmoprotectants and reinforce antioxidant defenses under drought, salinity, and temperature extremes [53]. Collectively, these coordinated microbial effects on osmoprotection, redox homeostasis, and hormonal regulation enable plants to maintain metabolic stability under abiotic stress.

### 2.4. Immune Modulation and Enhanced Stress Tolerance

Beyond growth promotion, PGPR and PGPF act as modulators of plant immune responses. Through the production of signaling molecules, microbial metabolites, or cell-wall components, these organisms prime defense pathways that bolster resistance to both biotic and abiotic stressors [54,55]. Fungi and bacteria may upregulate genes involved in systemic resistance, activate phenylpropanoid metabolism, increase lignification, and stimulate the production of antimicrobial compounds, all contributing to reduced disease severity and improved plant vigor [56,57,58]. Through these mechanisms, PGPR and PGPF collaboratively orchestrate a physiological state in which growth and defense are co-optimized rather than antagonistic.

### 2.5. Functional Integration Across Microbial Groups

Although often discussed separately, PGPR and PGPF operate within a shared functional landscape in which their activities complement and reinforce one another. Bacterial contributions to nutrient mobilization, phytohormone synthesis, and redox modulation interact with fungal effects on root-system development, osmotic adjustment, and immune priming, producing synergistic benefits that exceed those obtained from single-microbe inoculations. Through these combined actions, microbial communities collectively enhance root expansion, biomass accumulation, nutrient uptake efficiency, oxidative stress control, and immune readiness. This integrated network of microbial functions ultimately shapes a regulatory and metabolic environment that enables plants to sustain growth and maintain resilience under a wide array of environmental stressors [59].

Functional integration among plant-associated microbial groups extends beyond the additive effects of individual taxa and emerges from coordinated metabolic, ecological, and signaling interactions within the rhizosphere and endosphere. Bacteria, fungi, microalgae, and archaea frequently occupy complementary niches, enabling cross-feeding of metabolites, cooperative nutrient cycling, and stabilization of microbial communities under fluctuating environmental conditions. For instance, bacterial nitrogen fixation and phosphorus solubilization can synergize with fungal hyphal networks that enhance spatial nutrient redistribution, while microalgae contribute organic carbon and oxygen that support heterotrophic microbial activity. At the same time, archaeal involvement in nitrogen and carbon turnover can influence rhizosphere redox dynamics, indirectly shaping the activity of other microbial partners. These interactions are further coordinated through chemical signaling and shared stress-response pathways, allowing multi-kingdom microbial consortia to respond collectively to abiotic stress and to modulate plant physiological responses more effectively than single microbial groups. Thus, functional integration should be viewed as an emergent property of microbial community organization, driven by metabolic complementarity and environmental filtering rather than by isolated microbial traits [10,60,61].

Plant growth-promoting bacteria and fungi play central roles in plant development and stress mitigation, exhibiting phenotypic traits associated with plant growth performance and tolerance to environmental constraints, as summarized in Table 1.

### 2.6. Microalgae and Archaea as Key Contributors to Plant Performance and Stress Resilience

Microalgae, much like bacteria and fungi, represent an important component of the soil microbiota and contribute substantially to plant growth and stress mitigation [77]. These photosynthetic microorganisms, encompassing eukaryotic groups such as green algae and diatoms, as well as prokaryotic cyanobacteria, play versatile ecological roles that influence soil fertility and plant physiology. Their activities include carbon dioxide fixation, exopolysaccharide secretion that improves soil structure, nutrient mobilization, and the synthesis of phytohormones capable of stimulating plant development. Cyanobacteria, in particular, form heterocysts that specialize in atmospheric nitrogen fixation and facilitate phosphorus solubilization, enhancing the supply of essential nutrients to plants [78]. Notably, soils inoculated with microalgae exhibit markedly reduced nitrogen losses, approximately 7% compared to up to 50% under chemical fertilization, demonstrating their capacity to improve soil nitrogen retention [21].

Interactions between microalgae and bacteria further amplify these benefits through synergistic physiological and metabolic exchanges [21,79]. Central to this relationship is the phycosphere, a microenvironment enriched in proteins and carbohydrates surrounding algal cells, which varies in size depending on the composition and abundance of algal exudates and can either inhibit or stimulate neighboring microorganisms [80]. Microalgae supply oxygen, dissolved organic carbon, and calcium carbonate that promote bacterial proliferation, while bacteria reciprocate by providing key micronutrients such as iron, macronutrients including nitrogen and phosphorus, and metabolites such as carbon dioxide, vitamins, amino acids, and phytohormones, all of which support microalgal growth. These reciprocal exchanges enhance microbial activity and crop productivity by 5–25% and contribute to more efficient nutrient cycling and soil performance [21,81]. Gene transfer, nutrient exchange, and signal transduction pathways, shaped by biotic and abiotic factors, further highlight the role of microalgae as indirect promoters of plant growth and soil health [79].

Together, these algae–bacteria interactions exemplify how plant-beneficial effects emerge from multi-partner microbial networks rather than isolated organisms. Unlike single-strain inoculants, consortia can integrate complementary functional traits related to nutrient mobilization, stress mitigation, and signaling, while also improving ecological resilience through functional redundancy and cooperative interactions. Such multi-species assemblages are particularly relevant under climate change scenarios, where fluctuating abiotic stresses can compromise the performance of individual strains [82,83].

Archaea share similar ecological importance within the plant microbiome, functioning alongside fungi, bacteria, and microalgae as influential drivers of plant growth and resilience [84]. They exhibit multiple plant growth–promoting traits, including phosphorus solubilization, production of IAA, synthesis of siderophores, and participation in nitrogen and carbon fixation. These functions translate into clear phenotypic improvements: archaeal inoculation can stimulate root system development by increasing both primary and lateral root elongation and enhancing root hair density, resulting in improved water and nutrient uptake efficiency. Such root modifications support enhanced shoot development, including increases in stem length, leaf area, leaf number, and overall biomass accumulation [85,86].

Archaea also play a significant role in enhancing plant tolerance to environmental stressors. Through the production of phytohormones, osmolytes, and antioxidant enzymes, they help mitigate drought, salinity, temperature extremes, and heavy metal toxicity, leading to improved photosynthetic performance, reduced oxidative damage, and maintenance of cellular homeostasis under adverse conditions [85,87]. Their influence on nutrient cycling, particularly via ammonium production and carbon fixation, further supports plant growth in nutrient-poor soils [88]. Collectively, archaeal activity promotes larger root and shoot systems, enhanced leaf development, greater biomass accumulation, and improved stress resilience, underscoring their potential as valuable microbiome members in sustainable agriculture [85,86,87,89].

In addition to bacteria and fungi, plant-associated microalgae and archaea have emerged as relevant contributors to plant performance under diverse environmental conditions, as summarized in Table 2.

**Figure 1:**
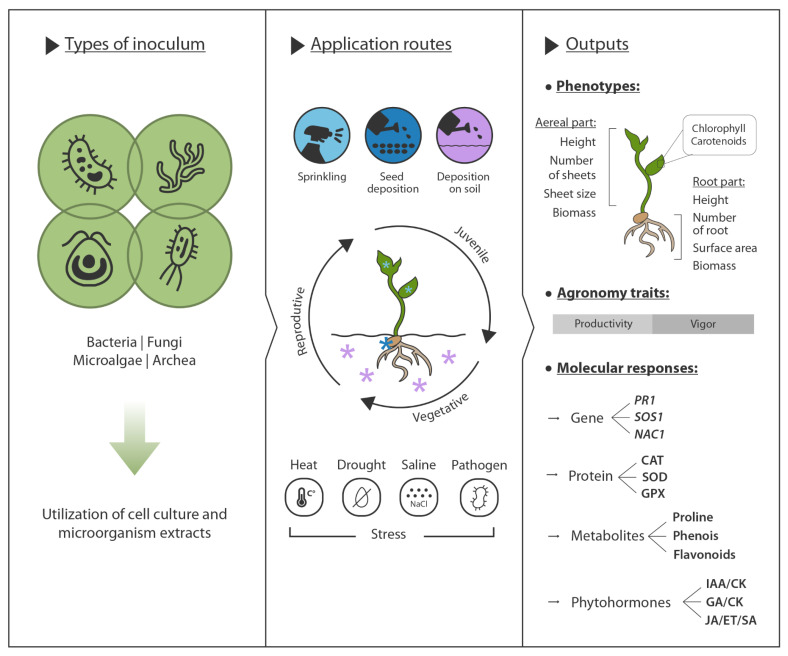
Schematic overview of how PGPMs are used to improve crop performance. Different types of inocula, bacteria, fungi, microalgae and archaea, are applied as cell cultures or microbial extracts and can be delivered by foliar spraying, seed coating or soil/substrate drenching at juvenile, vegetative or reproductive stages. Under multiple stresses (heat, drought, salinity and pathogens), these inoculations modulate plant outputs at three levels: (i) phenotypes, including shoot and root growth, leaf number and size, root surface area, biomass and pigment content (chlorophylls, carotenoids); (ii) agronomic traits, such as productivity and vigor; and (iii) molecular responses, comprising changes in gene expression (e.g., *PR1*, *SOS1*, *NAC1*), antioxidant enzymes (CAT, SOD, GPX), stress-related metabolites (proline, phenolics, flavonoids) and phytohormone balance (IAA/CK, GA/CK,/ET/ salicylic acid (SA) and jasmonic acid (JA)).

Together, microalgae and archaea complement the functions of bacteria and fungi within the plant microbiome. Their unique metabolic capacities, ecological interactions, and contributions to nutrient cycling and stress mitigation position them as integral partners influencing plant growth, productivity, and resilience across diverse agroecosystems. Bacteria, fungi, microalgae, and archaea collectively enhance plant performance by facilitating nutrient solubilization, producing phytohormones, synthesizing siderophores, fixing nitrogen and carbon, and inducing antioxidant and osmotic responses Figure 1. A detailed understanding of the molecular mechanisms underlying these interactions, including the genes involved, provides a strong foundation for the development of next-generation biofertilizers and biostimulants capable of maximizing plant growth, productivity, and health across diverse ecosystems.

## 3. Integrated Mechanisms Induced by Plant–Microorganism Interaction Under Biotic and Abiotic Stress Conditions

This section discusses the principal integrated mechanisms underpinning these coordinated responses, with a focus on genetic regulation and physiological adaptations.

### 3.1. Plant Immune Responses

Plants rely on a highly adaptable immune system that enables them to distinguish and respond to both pathogenic and beneficial microorganisms. The initial stage of this process involves the perception of microbe- or damage-derived signals, including pathogen-associated molecular patterns (PAMPs), microbe-associated molecular patterns (MAMPs), and damage-associated molecular patterns (DAMPs). These molecules are recognized by pattern recognition receptors (PRRs) located at the plant cell surface, activating early defense signaling [97,98]. Although these pathways have historically been described in the context of plant–pathogen interactions, mounting evidence indicates that PGPMs can engage the same recognition and signaling modules, eliciting responses that support beneficial associations rather than full defensive activation.

Upon activation of PRRs, plants initiate basal immunity, which in pathogenic encounters may progress to more specialized responses such as effector-triggered immunity. However, in the presence of PGPMs, these pathways are often modulated to generate a refined physiological state that enhances resilience without incurring the substantial metabolic costs typically associated with complete immune activation. Core components of plant immunity, including controlled bursts of ROS, phosphorylation cascades involving mitogen-activated protein kinases (MAPKs), and the regulated accumulation of phytohormones such as JA, ET, and SA, are mobilized at moderate levels, preparing the plant for future challenges while allowing growth to proceed [99,100].

Within this flexible immune framework, two major systemic states exemplify how plants recalibrate their defenses in the presence of beneficial microbes: Induced systemic resistance (ISR) and systemic acquired resistance (SAR).

ISR is commonly triggered by beneficial bacteria and fungi and is predominantly regulated through JA- and ET-mediated signaling. Instead of promoting immediate production of defense proteins, ISR establishes a primed physiological state in which the plant responds faster and more robustly to subsequent biotic or abiotic stress. This priming involves modulation of transcription factors and shifts in secondary metabolism that reinforce the plant’s defensive capacity [101].

SAR is traditionally associated with SA accumulation and the induction of pathogenesis-related (PR) proteins but can also be indirectly influenced by PGPMs. Beneficial microbes may modulate ROS dynamics, redox signaling, or hormone balance in ways that enhance the plant’s systemic resilience to stress. These interactions create hybrid immune states that combine elements of SAR and ISR, improving both defense responsiveness and physiological performance [99,101,102].

The PGPMs regulate plant immunity through mechanisms that intersect both ISR and SAR, but do so in a coordinated and context-dependent manner. Rather than activating ISR or SAR as isolated pathways, PGPM-derived signals influence the amplitude, timing, and hormonal integration of these defense responses, shaping downstream gene expression and defense readiness according to microbial identity, environmental conditions, and plant physiological status. In this framework, ISR and SAR should be viewed as interconnected components of a broader immune regulatory network modulated by beneficial microorganisms, in which JA, ET, and SA-associated pathways are dynamically integrated to optimize defense effectiveness while minimizing metabolic costs. This synthesis clarifies how PGPM-mediated immune regulation operates not through pathway exclusivity, but through balanced coordination between ISR- and SAR-associated responses [103,104,105].

Recent advances in multi-omics technologies have deepened our understanding of how PGPMs orchestrate these immune adjustments. Transcriptomic studies reveal that beneficial microorganisms reprogram the expression of genes involved in hormonal metabolism, defense priming, and oxidative balance, often generating transcriptional states distinct from those induced by pathogens [106]. Proteomic and metabolomic analyses have further shown that these transcriptional changes correspond to measurable shifts in protein abundance and metabolite accumulation associated with adaptation, resistance, and growth promotion [107,108].

In parallel, metagenomic and metatranscriptomic approaches have elucidated how PGPMs influence the composition and function of plant-associated microbiomes, while biochemical assays quantifying phytohormones, ROS, and antioxidant enzymes provide functional validation of these molecular adjustments [109]. Together, these integrated findings demonstrate that PGPM-mediated immunity arises not from the activation of a single pathway but from the coordinated remodeling of multiple gene networks that balance defense with growth.

Overall, plant immunity should be viewed as a dynamic regulatory system in which defense and development are continuously recalibrated by beneficial microbiome members. PGPMs do more than trigger immune pathways; they modulate their intensity, timing, and integration, enabling plants to maintain growth while enhancing resilience to biotic and abiotic stresses [110].

### 3.2. Plant Developmental and Defense Responses

A growing body of experimental work provides mechanistic support for the diverse pathways through which PGPMs enhance plant performance. For instance, studies with *B. subtilis* illustrate how bacterial colonization modulates host developmental and defense responses. In tomato, this bacterium promoted root elongation, consistent with microbially driven auxin signaling, and upregulated auxin transport-associated genes such as SIPIN6 and SILAX4 [111]. Simultaneously, *B. subtilis* inhibited the mycelial growth of soilborne pathogens, including *R. solani*, *Pythium ultimum*, and *F. oxysporum*. Transcriptomic profiling revealed coordinated activation of JA and ET-responsive genes (LOXD, CHI3, PAL) together with salicylic acid-dependent defense genes (PR-1A, GLUA), indicating that SA, JA, and ET signaling pathways act synergistically to suppress infection [112].

Comparable effects have been observed for *Bacillus thuringiensis* in Chinese cabbage (*Brassica campestris* L.) infected by *Sclerotinia sclerotiorum*. Exopolysaccharides produced by the bacterium acted as MAMPs, initiating ISR-like responses marked by elevated expression of HEL and PDF1.2, whereas the SA-associated transcription factor WRKY70 remained unchanged [113]. In maize, the plant growth-promoting bacteria (PGPB) *E. cloacae* enhanced plant height and photosynthetic activity during the *F. oxysporum* challenge. These improvements were associated with increased levels of IAA and GA, improved iron and ammonium availability, and induction of PR1, a marker of SAR [101].

PGPMs also mitigate viral diseases, which interfere with photosynthesis, protein synthesis, nutrient transport, and oxidative balance [14,114]. The manipulation of plant-associated microbiomes has emerged as a particularly promising strategy for inducing virus resistance, as reviewed in the context of next-generation biotechnological solutions [26]. In *C. annuum* L. infected with groundnut bud necrosis virus, *Bacillus amyloliquefaciens* enhanced both SA and JA defense pathways, increasing expression of NPR1, PAL, PO, SAR8.2, and JA-associated genes such as PDF, LOX, and EAS1, the latter encoding epi-aristolochene synthase involved in capsidiol biosynthesis [55]. Likewise, *Streptomyces pactum* reduced Tomato yellow leaf curl virus severity by inducing the SA-responsive gene PR-1a, the JA-associated gene SIPI-II, and viral resistance genes including SIVRSLip and SIPER1. Enhanced activity of ribonucleic acid (RNA) interference machinery (DCL2, AGO1, RDR6) further reinforced antiviral defenses [115]. A PGPF-based treatment for tomato mosaic virus also upregulated phenylpropanoid and flavonoid biosynthesis, increasing expression of PAL1, transcription factors AN1 and AN2, and HQT, a key gene in chlorogenic acid production [116].

PGPM-based biocontrol can also involve antimicrobial metabolites. Bacteriocins, extracellular antimicrobial peptides produced by rhizobacteria, disrupt pathogen membranes, leading to leakage of cellular contents [14]. Their structural and functional diversity arises from chromosomal or plasmid-encoded genes and tight environmental regulation [117,118]. Increasingly, bacteriocins are being investigated as potential antibiotic alternatives in mammals [119]. A notable example is a bacteriocin from *Leuconostoc mesenteroides*, which inhibited *Xylella fastidiosa* by destabilizing its membrane, ultimately causing loss of cellular integrity and cell death [120,121].

Siderophores, iron-chelating secondary metabolites produced by fungi, bacteria, and other microorganisms, also contribute to pathogen suppression [122,123]. In rice infected by *Magnaporthe oryzae*, siderophore-producing *Pseudomonas putida* reduced disease severity while enhancing root and shoot growth. Upregulation of OsPR1.1, OsNPR1, and OsPDF2.2 indicated activation of SA-associated resistance pathways [124].

Microbial volatile compounds (MVCs) extend PGPM influence by activating JA and ET-mediated ISR pathways and stimulating transcription factors such as ERF and NPR, which regulate PDF1.2 and PR1 [125]. In tomato, dimethyl disulfide (DMDS) inhibited the growth of *Sclerotinia minor* and upregulated PR1 and PR5, consistent with SA-mediated SAR activation [126]. DMDS produced by *Bacillus cereus* also suppressed *B. cinerea* Pers. and *Cochliobolus heterostrophus* in tobacco and maize [127].

### 3.3. Plant Responses to Abiotic Stresses

Substantial evidence also supports the role of PGPMs in mitigating abiotic stresses. Drought induces membrane damage, osmotic imbalance, electrolyte leakage, and the accumulation of ROS, often leading to growth suppression [128]. In sugarcane, *Bacillus megaterium* alleviates drought stress by promoting root development linked to auxin production, reducing H_2_O_2_ and MDA levels, and enhancing antioxidant enzyme activities, CAT and ascorbate peroxidase (APX). The bacterium also modulated carbon assimilation genes, including RBC-L, PEPC, and SPS, suggesting a microbial role in restructuring metabolic energy allocation during water limitation [129]. In millet, *Shewanella putrefaciens* and *Cronobacter dublinensis* increased drought tolerance by upregulating key genes involved in ABA, GA, and IAA biosynthesis (SbNCED, SbGA20ox, SbYU) [130].

PGPMs also enhance tolerance to salinity, which disrupts ionic homeostasis and impairs plant development [131]. In soybean, inoculation with the PGPF *Bipolaris* sp. increased shoot and root biomass, chlorophyll content, and the accumulation of osmolyte-forming organic acids such as citric and malic acid. Gene expression analyses showed downregulation of GMNARK and upregulation of GMAKT2, collectively improving K^+^ transport and restoring ionic balance [53]. In wheat, *B. megaterium*, *Bacillus tequilensis*, and *P. putida* improved chlorophyll content, reduced electrolyte leakage and water loss, and upregulated SOS1 and SOS4, both central to salinity tolerance [54].

Soil alkalinity further challenges plant performance by restricting nutrient availability, impairing water uptake, and generating oxidative stress [132]. Under alkaline conditions, *Chlorella* sp. enhanced tolerance in *A. thaliana* by increasing expression of antioxidative genes such as GPX1 and CAT2, both of which detoxify H_2_O_2_ [133]. Temperature extremes, particularly cold or heat, also threaten plant viability. High temperatures damage membranes and enzymes, while low temperatures induce osmotic stress, ice crystal formation, and oxidative stress [106]. In tomato exposed to cold, inoculation with *Trichoderma harzianum* increased expression of NAC1, a regulator of defense-related transcription; TAS14, which encodes a dehydrin involved in stress protection; and P5CS, a key enzyme in proline biosynthesis that facilitates osmotic adjustment [134].

### 3.4. Genetic Determinants of Plant–Microbe Compatibility

**Figure 2:**
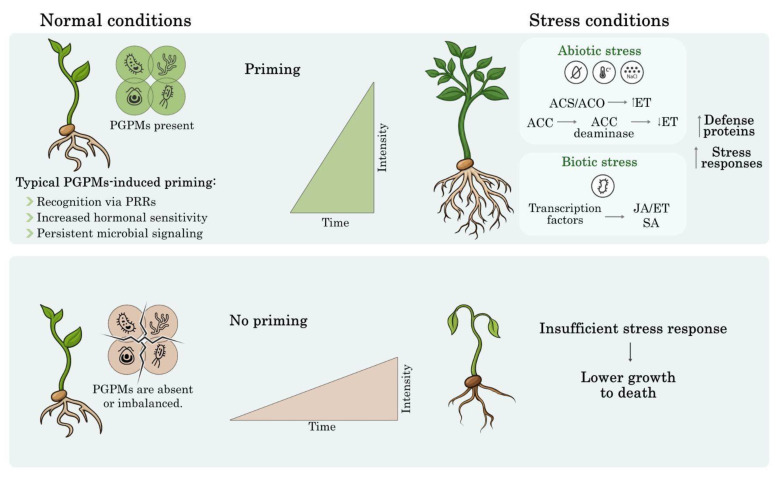
Priming mechanisms induced by PGPMs under biotic and abiotic stress conditions. The presence of PGPMs triggers a priming state through recognition via PRRs, increased hormonal sensitivity, and persistent microbial signaling. Upon stress exposure, primed plants exhibit a faster and more robust response, characterized by the upregulation of defense proteins and fine-tuning of hormonal pathways such as JA, ET, and SA. Specifically, under abiotic stress, the enzyme ACC deaminase plays a crucial role in lowering inhibitory ET levels. In contrast, the absence or imbalance of PGPMs leads to an insufficient stress response, resulting in stunted growth or plant death. The central diagrams compare the intensity of the defensive response over time in both scenarios.

Overall, genetic variation in both plants and microorganisms plays a central role in determining the effectiveness of symbiotic interactions by shaping compatibility, stability, and responsiveness under diverse environmental conditions Figure 2. Such variation influences how plants perceive and integrate microbial signals, regulate defense thresholds, and coordinate hormonal, metabolic, and redox responses, while microbial genetic diversity governs colonization efficiency, persistence, and functional expression in the rhizosphere. Consequently, the consistency and efficacy of beneficial plant–microbe associations emerge from integrated genetic regulation in both partners, which conditions the physiological and biochemical processes discussed above rather than relying on isolated gene effects [135].

At the host level, compatibility with beneficial microbes is largely dictated by the plant’s genetic architecture controlling recognition, immune modulation, and recruitment of microbial partners. Host genomes encode receptor kinases, such as LysM-type receptors that specifically bind microbial signaling molecules like Nod factors, initiating symbiosis signaling cascades in legume–rhizobium associations. Genome-wide association studies and genetic diversity analyses have identified plant loci that regulate root exudate composition, immune receptor sensitivity, and developmental pathways, which in turn influence the recruitment and retention of microbial taxa in the rhizosphere microbiome. For example, genetic variation in genes involved in plant development and nutrient uptake can modulate the profile of root exudates, which serve as chemoattractants or repellents, thereby conditioning the assembly of compatible microbial communities. Such host genetic determinants act not only in early signal perception but also in shaping the ecological niche in which microbes must operate to establish mutualism [136,137].

In contrast, the genetic determinants of microbial compatibility consist of suites of symbiosis-associated genes that enable recognition, signaling, and functional integration with the host. In rhizobia, large symbiotic plasmids carry key gene clusters such as *nod*, *nif*, and *fix*, which are essential for signal production, host specificity, and nitrogen fixation. The diversity of these genes across bacterial strains explains part of the variation in host range and compatibility, as different allelic variants of symbiosis genes interact differentially with plant receptors. Beyond signaling, microbial genomes encode surface polysaccharides, effector proteins, and secretion systems that modulate attachment, immune evasion, and adaptation to the host environment, further influencing compatibility outcomes. Thus, compatible plant–microbe interactions are the result of a molecular dialogue regulated by host and microbial genetic elements that ensure coordinated recognition, colonization, and mutual benefit under specific ecological contexts [138,139].

## 4. Genetic Engineering of PGPMs to Enhance Tolerance to Biotic and Abiotic Stresses

Understanding the genes involved in plant responses to biotic and abiotic stress, as modulated by PGPMs during plant-microbe interactions, is crucial for the development of novel biotechnological strategies aimed at improving crop resilience and productivity. The genetic engineering of microorganisms enables precise genomic manipulation to express genes required for specific functions, including those involved in the production of bioactive compounds, phytohormones, and stress-mitigating metabolites. A promising approach within this field involves engineering microorganisms to optimize the expression of key genes responsible for mitigating both biotic and abiotic stresses in plants, thereby enhancing their growth, tolerance, and overall fitness under adverse environmental conditions [140].

Microbial genetic modification research has mainly focused on bacteria because of their simple genomes, rapid replication, and well-characterized metabolism. The process involves identifying and isolating a target gene, inserting it into an expression vector, and introducing the recombinant deoxyribonucleic acid (DNA) into the host cell through methods such as transformation, transduction, or electroporation. Selectable markers, typically antibiotic resistance genes, enable the identification of transformed cells, while gene expression and function are confirmed through molecular and phenotypic assays [140,141].

For instance, in the context of biotic stress mitigation, the phlD and phlACB genes from *Pseudomonas protegens* Pf-5 and *Pseudomonas* sp. G22 has been amplified to promote constitutive expression of 2,4-diacetylphloroglucinol (2,4-DAPG), a well-known antifungal secondary metabolite, in *Pseudomonas* sp. WS5, a diazotrophic and endophytic bacterium capable of colonizing plant tissues. This engineered strain was subsequently inoculated into rice (*O. sativa* L.), sorghum (*Sorghum bicolor* L.), and wheat (*T. aestivum* L.) plants challenged with the fungal pathogens *M. oryzae* and *R. solani*. The constitutively produced 2,4-DAPG exhibited potent antifungal activity, significantly reducing disease severity while simultaneously promoting plant growth through indirect mechanisms, including enhanced nutrient uptake, stimulation of root architecture, and modulation of stress-responsive pathways [142]. Such studies highlight the potential of genetically engineered PGPMs to serve as dual-function bioinoculants that not only control pathogens but also improve plant performance under stress, representing a key innovation for sustainable agriculture and crop protection strategies.

Fungi can also be genetically modified to promote plant development. Unlike bacteria, which have more standardized transformation protocols, genetic modification in fungi requires species-specific optimization, with significant variation among methods [143]. One of the most widely used strategies is protoplast-mediated transformation, which involves the removal of the fungal cell wall through the action of enzymes such as glucanases. The protoplasts are then maintained in a solution containing calcium ions, which increase plasma membrane permeability, and osmotic stabilizers, which prevent cell lysis due to osmotic shock. Under these conditions, the DNA of interest can be incorporated into the cell. Following this process, the protoplasts regenerate their cell walls, and, similarly to bacterial transformations, selectable markers such as antibiotics are used to allow the growth of only the transformed fungi [144].

The fungus *Trichoderma hamatum* was genetically modified to act as a biocontrol agent against the fungal pathogens *S. sclerotiorum* and *R. solani*, which impair the growth of lettuce (*L. sativa* L.). A mutation was introduced in the NAG gene, which encodes the enzyme N-acetyl-β-D-glucosaminidase. This enzyme is involved in the degradation of chitin, a polysaccharide present in the fungal cell wall. The genetic modification contributed to pathogen suppression and improved phenotypic traits in the infected plants [145].

Building upon the roles of PGPBs and PGPFs in promoting plant growth and mitigating biotic stresses, genetic engineering of PGPMs has emerged as a powerful strategy to enhance plant resilience against abiotic stresses. For example, in peanut (*Arachis hypogaea* L.), the PGPB *Mesorhizobium* sp. was genetically modified to enhance trehalose accumulation through overexpression of the otsA gene, aiming to alleviate salt stress. Trehalose is a disaccharide that protects mitochondria from oxidative damage, helps maintain plasma membrane integrity, and functions as a stress response regulator. Inoculation with the genetically modified microorganism improved plant phenotypes under stress conditions, including higher root nodule numbers, increased dry mass and plant height, and significantly elevated activity of antioxidant enzymes such as MDA, SOD, and POD [146].

Genetic engineering aims not only to promote plant development but also to enhance the adaptation of PGPB to novel soil microbiomes, addressing the various challenges associated with the survival of these associations. In some cases, PGPB may fail to colonize the rhizosphere due to competition for nutrients with other microorganisms, as root exudates vary among plant species, attracting diverse microbial communities. Additionally, abiotic factors such as temperature, pH, and moisture influence the establishment and persistence of these microorganisms [147,148]. Considering these limitations, genetic engineering can be employed to optimize PGPB adaptation to the soil and rhizosphere, thereby enhancing their ability to promote plant growth and improve crop productivity.

Phosphorus is an essential nutrient for living organisms, playing a critical role in energy production in the form of adenosine triphosphate (ATP) and in the composition of nucleic acids [149]. To enhance the solubilization of insoluble phosphorus, the PCC gene, which encodes the enzyme phosphoenolpyruvate carboxylase, was overexpressed in fluorescent pseudomonads to increase the supply of oxaloacetate, a precursor in the biosynthesis of organic acids involved in phosphate solubilization [150]. As previously discussed, IAA synthesis is a bacterial strategy for root colonization, as increasing the contact surface area enhances the likelihood of bacterial association [151]. To induce higher IAA production, the PGPB *Cupriavidus pinatubonensis* was engineered with a mutation in the iaaM gene, which encodes tryptophan monooxygenase. This enzyme uses the amino acid tryptophan as a substrate, converting it into indole-3-acetamide, which is subsequently transformed into IAA [152].

Recent advances in plant–microbiome research increasingly combine synthetic microbial consortia (SynComs) with genome editing technologies to enhance stress resilience in a predictable and targeted manner. SynComs are rationally assembled communities composed of functionally complementary microbial strains selected for their stability and coordinated effects on plant nutrition, phytohormone signaling, redox homeostasis, and immune regulation. The efficacy of these consortia is strongly influenced by host-driven microbiome assembly processes, particularly the composition of root exudates that act as selective chemical cues for microbial recruitment and activity. In this context, clustered regularly interspaced short palindromic repeats (CRISPR) based genome editing emerges as a powerful tool to fine-tune plant traits that govern microbiome compatibility, including genes involved in exudate biosynthesis, immune recognition, and stress signaling pathways. By modifying host genetic determinants that shape microbial colonization and function, CRISPR technologies can enhance SynCom establishment, persistence, and functional output under abiotic and biotic stress conditions. The integration of SynCom design with host genome engineering thus represents a next-generation strategy for microbiome-assisted crop improvement, enabling precision manipulation of plant–microbe interactions while reducing reliance on chemical inputs and supporting sustainable agricultural systems [153,154,155]. This approach aligns with broader frameworks for engineering the plant microbiome to enhance resilience against biotic stresses, including viral diseases [26].

The integration of these genetic strategies complements the phenotypes and mechanisms observed, which similarly contribute to root system architecture, leaf area expansion, biomass accumulation, and stress resilience through phytohormone provision, osmolyte accumulation, and immune modulation [37,46,134]. These findings underscore the versatility of PGPMs, both naturally occurring and genetically enhanced, in promoting plant development, improving nutrient acquisition, and conferring tolerance to biotic and abiotic stresses. By leveraging microbial engineering, it is possible to design tailored bioinoculants that optimize plant growth and resilience across diverse environmental conditions, representing a promising avenue for sustainable agriculture and increased crop productivity. The focus lies on bioinoculants capable of enhancing plant stress resistance. Within this framework, a dual-method cross-validation strategy is applied, representing a robust analytical approach widely used in fermentation science to reliably identify genes involved in stress-resistance mechanisms in plant-associated microorganisms [156].

However, it is important to note that the effectiveness of a microbial inoculant is strongly influenced by the host plant, the composition of the rhizosphere microbiome, and prevailing environmental conditions. In this context, engineering broad-spectrum microorganisms capable of interacting with multiple plant species represents a promising strategy, particularly when targeting conserved genetic traits associated with plant growth promotion, including nitrogen fixation, siderophore production, and phosphate solubilization. Such modifications may enhance functional stability across diverse hosts and environments, thereby improving the consistency and applicability of microbial technologies in agriculture [132,157,158].

From an environmental safety perspective, the deployment of genetically modified microorganisms (GMMs) in agriculture requires careful evaluation beyond their agronomic benefits. Potential risks include unintended effects on native microbial communities, horizontal gene transfer, and long-term ecological shifts resulting from the introduction of engineered traits into complex soil ecosystems. Environmental conditions such as climate variability, soil heterogeneity, and microbial competition may further influence the behavior, persistence, and ecological impact of GMMs outside controlled settings. Consequently, risk assessment frameworks must account for context-dependent interactions between engineered microorganisms, host plants, and resident microbiota. Regulatory oversight and biosafety guidelines are, therefore, essential to ensure that microbiome-based biotechnologies align with sustainable agriculture goals while minimizing ecological disruption. Integrating molecular containment strategies, post-release monitoring, and environment-specific risk evaluation represents a critical step toward the responsible use of genetically engineered microbes in agroecosystems [159,160,161].

## 5. Conclusions

The convergence of molecular biology, microbial ecology, and biotechnology has reshaped our understanding of plant–microorganism partnerships, establishing them as a strategic foundation for agricultural sustainability. The studies compiled in this review demonstrate that beneficial interactions with bacteria, fungi, microalgae, and archaea are not merely supportive but essential for plant resilience, driving phenotypic improvements in growth, yield, and stress tolerance through well-defined genetic and molecular mechanisms. The genetic bases of these symbioses, ranging from microbial genes involved in phytohormone synthesis, nutrient solubilization, antimicrobial compound production, and signaling, to plant genes associated with immune priming and systemic resistance, provide a clear blueprint for biotechnological innovation.

Within this framework, the engineering of plant growth–promoting microorganisms emerges as a transformative frontier. Enhancing traits such as antifungal metabolite production, stress mitigation capacity, or rhizosphere competence illustrates the potential to amplify and tailor beneficial microbial functions. However, fully realizing this potential requires overcoming the translational gap between laboratory findings and field performance, a challenge shaped by the dynamic interplay among plant genotype, native soil microbiome, and environmental variability, all of which influence the consistency of bioinoculant efficacy. Future strategies must therefore prioritize context-adapted microbial formulations, synthetic microbial consortia with complementary functions, and plant cultivars optimized to recruit and sustain beneficial microbiomes.

Advancing toward effective integration of microbiome management into agricultural practice demands a systems-level perspective. This includes leveraging multi-omics tools for real-time monitoring of plant–microbe interactions, refining formulation and delivery technologies to ensure microbial survival and activity, and establishing regulatory frameworks that support the safe and widespread adoption of these biological innovations. Ultimately, harnessing plant–microbe partnerships is not simply an alternative approach but a necessary evolution toward a new agricultural paradigm. Translating mechanistic insights into scalable, field-ready applications will enable the development of cropping systems that are more resilient to biotic and abiotic stresses, less dependent on chemical inputs, and fundamentally aligned with ecological sustainability, an essential step toward securing the future of food production in a changing climate.

Despite the robust body of experimental evidence supporting the beneficial effects of PGPM under controlled conditions, scaling up these findings for field applications remains a major challenge. Variability in soil physicochemical properties, climate conditions, crop genotype, and native microbiota are important characteristics for the inoculant formulation. In addition, large-scale production, formulation stability, shelf life, and delivery methods substantially influence inoculant efficacy and cost-effectiveness. Regulatory frameworks governing bioinoculants, together with limited farmer adoption driven by variable outcomes and insufficient extension support, further constrain large-scale implementation. Addressing these barriers requires integrative field trials, environment-specific strain selection, and closer alignment between microbial ecology, agronomy, and regulatory science.

## Figures and Tables

**Table 1:** Effects of plant growth-promoting bacteria and fungi in plant stress responses.

Microorganism(s)	Host Plant	Stress	Phenotype	Ref.
Bacteria				
*Pseudomonas fluorescens*	*Lycopersicon esculentum* Mill.	Fluoride stress	Increase in leaf area and root length; accumulation of soluble sugars, glycine betaine, proline, and total chlorophyll.	[62]
*Bacillus ginsengihumi* and *Bacillus atrophaeus*	*Lactuca sativa* L.	Drought	Increased IAA production, biomass, and nitrogen uptake.	[63]
*Stenotrophomonas maltophilia*, *Bacillus subtilis*, *Enterobacter hormaechei*, and *Staphylococcus epidermidis*	*L. esculentum* Mill.	Saline	Increased biomass, shoot and root size; enhanced antioxidant enzyme activity, proline, and chlorophyll.	[64]
*B. subtilis*, *Mesobacillus subterraneuse*, and *Brevibacillus parabrevis*	*Oryza sativa* L.	Drought, saline, and arsenic stress	Reduction in malondialdehyde (MDA); increased plant height and leaf number; enhanced activities of CAT and GPX.	[65]
*Bacillus safensis*	*Solanum lycopersicum* L.	Heat	Increased leaf area, flower and fruit number, biomass, photosynthetic pigments, leaf water content, and antioxidant enzyme activities.	[66]
*Enterobacter cloacae*	*O. sativa* L.	Saline	Effects related to phytohormone secretion and increased ACC deaminase activity, reducing ethylene levels.	[67]
*Rhizobium leguminosarum*	*Phaseolus vulgaris*	*Xanthomonas axonopodis*	Increased root biomass, pod and seed number, and reduced pathogen-induced symptoms.	[68]
*B. atrophaeus*, *Pseudomonas parafulva*, and *Trichoderma virens*	*Brassica napus* L.	*Plasmodiophora brassicae*	Increased root length, modulation of endogenous hormone levels, and secondary metabolites. The consortium of the three strains significantly reduced disease severity.	[69]
*Herbaspirillum seropedicae*	*L. esculentum* Mill.	*Xanthomonas euvesicatoria*	Increase in fresh biomass and shoot and root length. Reduction in disease incidence and a lower number of leaflets with symptoms.	[70]
*B. subtilis*	*Carica papaya* L.	*Erwinia mallotivora*	Reduced crown rot incidence in both susceptible and resistant papaya genotypes.	[71]
*P. fluorescens*	*Olea europaea* L.	*Verticillium dahliae*	In vitro antagonistic activity and in vivo reduction in disease symptoms caused by the fungus.	[72]
**Fungi**				
*Funneliformis mosseae*	*Capsicum annuum* L.	Saline and *V. dahliae*	Increased leaf water content, phenolic and antioxidant compounds, as well as enhanced biomass and shoot and root size.	[73]
*Acremonium alternatum*	*B. napus* L.	Saline	Decreased oxoglutarate, aspartate, hydrogen peroxide (H_2_O_2_), and superoxide. Increased defense compounds, including nervonic acid, brassinin, and phenolics.	[74]
*Periconia macrospinosa*, *Neocamarosporium goegapense*, and *Neocamarosporium chichastianum*.	*Hordeum vulgare* L.	Saline and drought	Increased biomass, greater shoot and root length, higher chlorophyll and proline content, and enzyme activities (CAT, POD, and SOD).	[75]
*Aspergillus chevalieri* and *Aspergillus egyptiacus*	*Vicia faba* L.	*Alternaria solani*	Increased production of siderophores, IAA, and antioxidant enzymes, with reduced MDA and hydrogen peroxide levels.	[58]
*Alternaria photistica*, *Penicillium buchaldii*, and *Aspergillus niger*	*L. esculentum* Mill.	*Meloidogyne incognita*	Increased levels of chlorophyll and carotenoids, total root carbohydrates, and shoot proteins in both healthy and infected plants.	[57]
*Aspergillus fumigatus* and *Rhizopus oryzae*	*L. esculentum* Mill.	*Fusarium oxysporum*	Reduced disease severity, increased leaf number, and enhanced shoot and root length.	[56]
*Purpureocillium lilacinum*	*Solanum melongena* L.	*Mucor piriformis*, *Trichothecium roseum*, *Rhizoctonia solani*, and *V. dahliae*	Increased chlorophyll content, germination rate, and root length, with reduced disease incidence.	[76]

**Table 2:** Effects of plant growth-promoting microalgae and archaea on plant stress responses.

Microorganism(s)	Host Plant	Stress	Phenotype	Ref.
Microalgae				
*Asterarcys quadricellularis*	*Cucumis melo* L.	Saline	Increase in fresh and dry biomass, shoot size, photosynthetic pigments, total amino acids, total sugars, antioxidant enzymes, and phenolic compounds.	[90]
*Coelastrella* sp.	*Ocimum basilicum* L.	Drought and waterlogging	Increase in germination rate, vigor index, root length, and fresh and dry biomass. Reduction in lipid peroxidation and enhancement of SOD activity.	[91]
*Anabaena* sp., *Ecklonia* sp., and *Jania* sp.	*Botrytis cinerea*	*Fragaria* × *ananassa*	Reduction in infected area, sporulation capacity, and colony number.	[92]
*Chlorella vulgaris*	*Brassica oleracea* L.	Drought	Increased pigments, flavonoids, and reduced MDA.	[93]
*Azotobacter beijerinckii* and *Chlorella pyrenoidosa*	*Triticum aestivum* L.	saline-alkaline	Increased root growth and dry weight, improved N, P, and K uptake, and proline accumulation alleviating saline stress.	[94]
**Archaea**				
*Haloferax*	*Zea mays* L.	Cobalt stress	Reduced cobalt uptake, increased proline, sucrose, and phenolics, and enhanced antioxidant enzymes POX, CAT, and SOD.	[95]
*Haloferax*	*T. aestivum* L.	Cobalt stress	Increased sugars, organic acids, amino acids, and key enzymes, including proline, glutamine synthetase, and threonine synthase.	[96]
*Nitrosocosmicus oleophilus*	*Arabidopsis thaliana*	*Pectobacterium carotovorum* and *Pseudomonas syringae*	Regulation of molecules essential for Induced Systemic Resistance, such as benzothiadiazole.	[84]

## Data Availability

No new data were created for this review.

## Abbreviations

The following abbreviations are used in this manuscript:

2,4-DAPG	2,4-diacetylphloroglucinol
ABA	Abscisic acid
ACC	Aminocyclopropane carboxylic acid
ACO	Aminocyclopropane carboxylate oxidase
ACS	Aminocyclopropane carboxylate synthase
APX	Ascorbate peroxidase
ATP	Adenosine triphosphate
CAT	Catalase
CK	Cytokinins
CRISPR	Clustered regularly interspaced short palindromic repeats
DAMPs	Damage-associated molecular patterns
DMDS	Dimethyl disulfide
DNA	Deoxyribonucleic acid
ET	Ethylene
GA	Gibberellins
GMMs	Genetically modified microorganisms
GPX	Glutathione peroxidase
H_2_O_2_	Hydrogen peroxide
IAA	Indole-3-acetic acid
ISR	Induced systemic resistance
JA	Jasmonic acid
MAMPs	Microbe-associated molecular patterns
MAPKs	Mitogen-activated protein kinases
MDA	Malondialdehyde
MVCs	Microbial volatile compounds
NaCl	Sodium chloride
NU	United Nations
PAMPs	Pathogen-associated molecular patterns
PGPB	Plant growth-promoting bacteria
PGPF	Plant growth-promoting fungi
PGPM	Plant growth-promoting microorganisms
PGPR	Plant growth-promoting rhizobacteria
POD	Peroxidase
PR	Pathogenesis-related
PRRs	Pattern recognition receptors
RNA	Ribonucleic acid
ROS	Reactive oxygen species
SA	Salicylic acid
SAR	Systemic acquired resistance
SOD	Superoxide dismutase
SDGs	Sustainable Development Goals
SynComs	Synthetic microbial consortia
